# Annotations

**Published:** 1890-11-29

**Authors:** 


### Annotations.

**Lady Rosebery.**

In common with all who knew anything of Lady Rosebery, the nurses of England and Scotland, and more especially those who are associated with the Royal National Pension Fund, and with the Jubilee Institute, will mourn her premature death. Lady Rosebery was one of the patronesses of the Royal National Fund, and president of the Scottish Jubilee Institute of Trained Nurses. The loss of a good woman is a grief to all who may have known her, or come within the sphere of her influence. But when such a woman dies almost at the very beginning of her intelligent and beneficent career, the loss becomes a calamity. Lady Rosebery possessed, in an unusual degree, judgment, force of character, and kindness of heart. She had a purpose to live rightly and well, not for herself or her family only, but for her dependents, her neighbours, the mass of the people, and the State. Endowed by nature with gifts that please and inspire confidence, and inheriting from illustrious ancestors a great name and the power of wide beneficence, she was minded to preserve the traditions of her family, and to add lustre to the noble house of which she became a member by marriage. All those plans and purposes have been broken off by premature death. But Lady Rosebery has lived long enough to show to her friends and the world, of what kind of nature she was; and she has lived long enough to leave an example and a memory which will not only be followed and preserved by her own family and class, but by all sorts and conditions of women. The pen moves heavily as we strive to speak with fitting words and sympathetic feeling of those who are dead. They do not hear what we say; and if they did they would probably ask us to refrain from speech. But those who are still living, the husband, the children, the blood relations, and the near friends, may feel comforted by the knowledge that many of all ranks—all, indeed, who knew Lady Rosebery personally, or knew anything of her—ask to be permitted to share their sorrow and to express an affectionate reverence for the dead. Lady Rosebery belonged to an ancient race, whose record dates from the dawn of human story. In the order of the Divine government the leading members of that venerable and wonderful nation have found, not an asylum, but a home among the greatest, mightiest, and freest people of the present era. Still preserving their nationality and their venerable traditions, they yet become true citizens and patriots of their chosen country, dwelling peacefully with their fellow-men of another nationality, sharing the burdens and honours of empire, and mingling their blood with that of their adopted people. It is a strange and interesting, even a pathetic story. The East and the West, the old and the new, unite to mourn with Lord Rosebery at the grave of his dead wife; unite to mourn, but also to be consoled, and to thank God that, inasmuch as she was to die early, she yet lived long enough to leave a noble, a tender, and an enduring memory in the hearts of her family and of all who had any knowledge of her.

**A Word to the Governors of the London Hospital.**

Some of the officials of the London Hospital have been attacked by private persons. Those private persons have gained the ear of a popular journal. That popular journal has shown signs during the last fortnight of a purpose to take sides against the officials. The partiality of the journal in question was evidenced by its references to a paper read before the Hospitals Association by Mr. Burdett last week at St. Thomas's Hospital. The paper had for its object the consideration of the food, work, and recreation of hospital nurses. Those questions were discussed from the standpoint of nursing in general; and were illustrated in detail by references to the London Hospital, St. Bartholomew's, and a great many of the principal general hospitals both in London and the country. Incidentally Mr. Burdett referred to the recent dispute at the London Hospital, and expressed the emphatic conviction that those who would take the trouble to ascertain the facts as he had done would come to the conclusion that certain charges made against officials of the London were not only trivial in themselves, but entirely destitute of proof. Although this incidental reference was made to the affairs of the London, the greater part of the paper dealt in detail and with great fullness of information with the questions of nurses' food, work, and recreation. Notwithstanding this, the evening paper in question gave its readers to understand on the following day that Mr. Burdett had devoted his paper almost or quite exclusively to a defence of the officials of the London Hospital. Now that was a misrepresentation of the facts, and was quite unworthy of the character and position of the journal in question. Recent references by the same journal to an unfortunate incident which is said to have happened to an out-patient show that the same unfair animus still exists. It is to be anticipated, therefore, that if, by any unlooked for accident, the accusing party should snatch a vote at the governors' meeting which is to be held on the 3rd proximo, the very worst possible face will be put upon the fact not only by the accusers, but by the journal which so eagerly lends itself to their purposes. The plain truth is that the reputation and usefulness of the London Hospital are both endangered by the skilful and persistent blowing of a ridiculous bubble. The governors of the London Hospital are imperatively called upon at the present crisis to go to the meeting of the hospital next Wednesday at one o'clock and to do their duty. What is that duty? It is to demand that all the facts shall be fully brought before them; to insist that every charge made shall be proved; to distinguish between charges that are trivial and charges that are important; to refuse to be carried away by excitement; to give a solid, honest, and manly consideration to all the questions at issue; and to vote like fearless Englishmen according to their consciences. Every governor of the London Hospital should feel that duty demands his or her personal presence at the meeting, and the exercise of their best and most fearless judgment on all the resolutions which may then be proposed.

**Diphtheria at Croydon.**

Dr. Alfred Carpenter shows the zeal of an apostle in his efforts to make Croydon one of the healthiest towns in the kingdom. It has happened to him as it has happened to many apostles; his doctrines have been called "fads," and he himself has been voted a "nuisance." There is something to be learnt from the story of the sanitation of Croydon, as it is told by Dr. Carpenter in the morning papers. So long ago as 1865 Croydon suffered from an outbreak of typhoid fever. The drains were at fault, said Dr. Carpenter; and accordingly he reasoned and persuaded the local authorities with all his energy to get the drains put right. But why should a doctor pester a town council with arguments and contentions about town drainage? The doctor finds his profit in bad drains, not in good ones. When the drains are good the doctor may sit at home; there is little for him to do. But bad drains are gold mines to him. And yet Dr. Alfred Carpenter, and scores of others like him, get themselves disliked, and deprive themselves of work by their eagerness about the town drains. The philanthropy of the doctor overcomes his love of money and his desire for business success. But, should not a fact like this make even the most stolid of mayors and town councillors begin to think a little? These doctors cannot be mere "faddists"; they would not give offence, and lose patients and fees for the sake of fads that cannot be proved. The only reason why doctors are in such deadly earnest about drains and sanitation in general is because they know that they are dealing with stern and solid facts; facts that carry life and death within them. Dr. Carpenter in 1865 insisted, among other things, that the drains should be laid in concrete at Croydon. The object of this was to prevent the soaking of the ground with poisonous sewage in the event of accidental leaks. Ground soaked with sewage, it may be stated, becomes at once a bed of pestilence. But the local builders at Croydon were not accustomed to laying drains in concrete, and what they did not know could not, they thought, be worth knowing. So the doctor was worsted in the fight, and the stupid ones won the day. For that stupidity Croydon is now paying with the lives of its fathers and mothers, its sons and its daughters; and it has been so paying for the last quarter of a century. What ought to have been done twenty-five years ago will have to be done now; and probably at a greatly increased cost. All towns in which intelligent counsels have prevailed have been laying their drains in concrete for many years. The case of Croydon offers a warning and an example, not only to all sanitary authorities, but to every intelligent man and woman in the United Kingdom.

